# Pacing Strategy Affects the Sub-Elite Marathoner’s Cardiac Drift and Performance

**DOI:** 10.3389/fpsyg.2019.03026

**Published:** 2020-02-19

**Authors:** Véronique Louise Billat, Florent Palacin, Matthieu Correa, Jean-Renaud Pycke

**Affiliations:** ^1^IEA 3625/Institut des Sciences du Sport/I3SP, Université de Paris, Paris, France; ^2^Laboratoire de Mathématiques et Modélisation d’Evry, CNRS, Université Evry, Université Paris-Saclay, Evry, France

**Keywords:** running, endurance, Kendall, Strava, big data

## Abstract

The question of cardiac strain arises when considering the emerging class of recreational runners whose running strategy could be a non-optimal running pace. Heart rate (HR) monitoring, which reflects exercise intensity and environmental factors, is often used for running strategies in marathons. However, it is difficult to obtain appropriate feedback for only the HR value since the cardiovascular drift (CV drift) occurs during prolonged exercise. The cardiac cost (CC: HR divided by running velocity) has been shown to be a potential index for evaluation of CV drift during the marathon race. We sought to establish the relationship between recreational marathoners’ racing strategy, cardiac drift, and performance. We started with looking for a trend in the speed time series (by Kendall’s non-parametric rank correlation coefficient) in 280 (2 h30–3 h40) marathoners. We distinguished two groups, with the one gathering the large majority of runners (*n* = 215, 77%), who had a significant decrease in their speed during the race that appeared at the 26th km. We therefore named this group of runners the “fallers.” Furthermore, the fallers had significantly lower performance (*p* = 0.006) and higher cardiac drift (*p* < 0.0001) than the non-fallers. The asymmetry indicator of the faller group runners’ speed is negative, meaning that the average speed of this category of riders is below the median, indicating that they ran more than the half marathon distance (56%) above their average speed before they “hit the wall” at the 26th km. Furthermore, we showed that marathon performance was correlated with the amplitude of the cardiac drift (*r* = 0.18, *p* = 0.0018) but not with those of the increase in HR (*r* = 0.01, *p* = 0.80). In conclusion, for addressing the question of the cardiac drift in marathon, which is very sensitive to the running strategy, we recommend to utilize the cardiac cost, which takes into account the running speed and that could be implemented in the future, on mobile phone applications.

## Introduction

Every year, the New York, London, Berlin, and Paris marathons each attract around 30,000–50,000 adult runners of all levels. Most of these runners are recreational athletes. Many train alone and hope to progress by monitoring their heart rate and/or running speed. However, more and more studies showed that the self-pace exercise allows one to get the best performance. Indeed, it has been suggested that runners tend to adopt the same overall pacing strategy, even though several different strategies are available for events of different distances and durations. Hence, the most important factor in choosing a pacing strategy is knowledge of the endpoint of a particular event (St Clair Gibson et al., 2006). In line with this teleoanticipation hypothesis, the estimation of time limit corresponds to the perception of time when exercise at a constant power and a constant speed is performed until exhaustion (Coquart et al., 2012). However, the human self-paces his/her effort in real life, and notably in races from the 1,500-m middle-distance event upwards; we have used differential modeling of anaerobic and aerobic metabolism to show that runners continuously adjust their speed as a function of the remaining anaerobic capacity reserve (Billat V. et al., 2009). The latter finding demonstrated that runners are able to adjust their effort by varying their running speed according to perceived effort (Giovanelli et al., 2019; Molinari et al., 2019).

Every intensity zone of pacing corresponds to a specific effort perceived by the athlete (Kraemer et al., 2012). Indeed, the rate of perceived exertion (RPE) is closely related to the concept of exercise intensity, and more precisely, it is “the feeling of how heavy and strenuous a physical task is” (Borg, 1998). It does not take into account exclusively the workload but included many other factors that affect the performance (temperature, humidity, energy supply). Moreover, RPE is a key mediator in the regulation of the work rate (Tucker and Noakes, 2009). The importance of RPE in determining the self-selected exercise intensity (i.e., pacing) is underlined by authors (for details, see Abbiss and Laursen, 2008) who suggested that the brain processes a complex algorithm including peripheral feedback, previous experiences, and the remaining workload (Abbiss and Laursen, 2005; St Clair Gibson et al., 2006; Faulkner et al., 2008). Ceci and Hassmen (1991) showed that runners were able to self-adjust the running intensity at three different RPE values. These subjects adapted the speed in order to obtain an RPE of 11 (“light” on a 6–20 Borg scale) for 3 min, 13 (“somewhat hard”) for 11 min, and 15 (“hard”) for 5 min (17).

We recently found that recreational runners were able to adjust their acceleration every 4 s by asking them to do “soft,” “medium,” or “hard” accelerations (Billat et al., 2018). Molinari et al. (2019) showed that the runners maintained their speed in a steady-state manner during the submaximal stages (RPE 11 and 14) while increasing the intensity of their cardiorespiratory responses as the run continues. In contrast, our recreational runners maintained their $\dot{\text{V}}\,\text{O}2\,\text{max}$ during the maximal stage (RPE 17) by dropping their running speed. Thus, one can hypothesize that a runner uses the variation in speed (i.e., acceleration) as a marker to preserve the power of running-specific muscles – even when the cardiorespiratory variables are rising (Billat et al., 2018).

However, we still do not know how the runner associates a given RPE with speed vs. time. In perspective of the practical applications, we know that most runners train by monitoring their heart rate and running speed, rather than the perceived effort. To better understand the mechanisms of self-paced control in an ecological setting (such as track tests and trail races), there is a need to examine the speed variation and the pacing strategy of the recreational runners for being able to analyze their performance not only with the average pace but also with the pacing at each kilometer of the race.

Indeed, we hypothesize that this emerging class of recreational runners, whose running pace would be non-optimal, could have a higher cardiac cost increase during the race, especially after the half marathon. A prior study of our working group registered the cardiac output (CO) during a marathon in 14 recreational runners (3 h30 ± 45 min) and demonstrated that marathon performance was inversely correlated with an upward drift in the CO/speed ratio (mL of CO m^–1^) named the cardiac cost (Billat et al., 2012; Shimazu et al., 2013). This cardiac cost drift was due to the decrease in speed mainly after the half marathon while heart rate, stroke volume, and the CO were not significantly different between the first and the last 4 km (Billat et al., 2012). Furthermore, a recent study performed on big data available on Strava^®^, a social fitness network, has shown that 80% of recreational (2 h30–3 h40 min) marathoners had a negative distribution of their speed since it drops after the half race. In probability theory and statistics, skewness is a measure of the asymmetry of the probability distribution of a real-valued random variable about its mean. The skewness value can be positive or negative, or undefined. For a unimodal distribution, negative skew commonly indicates that the tail is on the left side of the distribution, and positive skew indicates that the tail is on the right side. In cases where one tail is long but the other tail is fat, skewness does not obey a simple rule. For example, a zero value means that the tails on both sides of the mean balance out overall; this is the case for a symmetric distribution, but can also be true for an asymmetric distribution where one tail is long and thin, and the other is short but fat. Hence, from a practical point of view, it means that they ran more distance (median speed) above their average speed (Billat et al., 2018). This was in opposition with best world performance marathons (Gebreselassie and Kipchoge races) (Billat et al., 2018).

When the runners wear a cardio frequency meter in addition to a GPS, the heart rate is also indicated in the runners’ Strava^®^ file. Therefore, it gives the possibility to highlight the relationship between speed strategy, heart dynamics, and performance in real racing conditions. Indeed, the marathon race is considered to demand extreme physical endurance and has provided a unique opportunity to study the limits of human thermoregulation for more than a century (Cheuvront and Haymes, 2001). Voluntary reduction in exercise intensity and/or duration is one of the most obvious behavioral thermoregulatory responses to hot environments and is done (at least partially consciously) in order to reduce heat production and the rate at which core body temperature rises (Tyler, 2019).

Therefore, in the present study, we sought to establish whether their racing strategy affects the sub-elite marathoner’s cardiac drift and performance. The aim of this study was then to check the hypothesis that, in recreational but already good marathoners (2 h30–3 h40), their racing strategy impacted their performance in relation to a possible cardiac drift.

## Materials and Methods

### Population

The 280 analyzed runs came from marathon runners who took part in the “Marathon de Paris” in 2018 (*n* = 140) or in the “Marathon de Berlin” in 2017 (*n* = 140). The chronometric performances were between 2 h30 and 3 h40 for each race. Every subject publicly shared his or her race data using the website Strava^®^, on which we proceeded to the gathering of the data. Therefore, given that the study used the Strava public data, we got neither the gender nor the age of the subjects.

### Experiment Protocol

#### Data Sampling

In order to have a constant performance sample, we gathered them from the fastest run (2 h30) to the slowest (3 h40), with an interval of 30 s between each runner.

#### Observed Variables

We had access to the running pace (time passed for each kilometer ran) and to the mean of the heart rate (HR) by kilometer. In our study, we did not consider the last 195 m of the run.

#### Computed Variables

The cardiac cost (CC) (which has a unit corresponding to the amount of heartbeat by meter ran) was computed with the mean of the heart rate (in b min^–1^) and the speed (in km min^–1^) by kilometer using the following formula:

$$\mathrm{Mean}\,\mathrm{cardiac}\,\mathrm{cost}=\frac{\mathrm{HR}\,\left(b\,\min^{-1}\right)}{\mathrm{speed}\,\left(m\,\min^{-1}\right)}/6000$$

### Statistical Study

#### Defining the Running Strategy by the Skewness of the Pace Distribution During the Race

In this study, we have characterized the running strategy by the skewness of the speed distribution during the race. The skewness was calculated from the number of kilometers ran above of the mean speed expressed in percentage of the 42 km of the marathon. In this study, we defined the running strategy as the skewness of the speed data by kilometer.

#### Modeling the Time Series Tendencies

We used Mann Kendall’s non-parametric test of trend on the speed, HR, and CC series. The result of this test, Kendall’s τ, between -1 and 1, gives us the statistical trend of the studied series. The statistic of Kendall’s τ is defined by the following formula:

$$\tau =\frac{2}{n\,\left(n-1\right)}\,\sum_{i<j}K\,\left(v_{i},v_{j}\right)$$

Like all the statistical tests, Mann Kendall’s trend test is read with a *p*-value. The closer to 0 this one is, the stronger the significance of the trend is. On the other hand, the closer to 1 this *p*-value is, the weaker the significance of the trend is.

In order to get the range Δ of this trend, we used the evolution coefficient below, where μ_0_ is the mean of the first 4 km and μ_1_ is the mean of the last 4 km.

$$\Delta =\frac{\left(\mu_{1}-\mu_{0}\right)}{\mu_{0}}\times 100$$

We chose to use the distance of 4 km because it represented nearly 10% of the marathon’s distance.

#### Determining a Heterogeneity Among Time Series

We used Pettitt’s non-parametric test, which is an adaptation of Mann–Whitney’s rank-based test, allowing to identify the time where a shift occurs. This statistical test is read with a *p*-value.

Pettitt’s statistic test is computed like the following:

We set:

$$D_{i\,j}=-1\,\text{if}\,\left(x_{i}-x_{j}\right)<0,D_{i\,j}=0\,\text{if}\,\left(x_{i}-x_{j}\right)=0,$$

$$D_{i\,j}=1\,\text{if}\,\left(x_{i}-x_{j}\right)>0$$

We then define:

$$U_{t,T}=\sum_{i=1}^{t}\sum_{J=i+1}^{T}D_{i\,j}$$

The alternative bilateral assumption of Pettitt’s test is defined by:

$$K_{T}=\max_{1\leq t<T}\left|U_{t,T}\right|$$

Applied to the time series of speed by kilometer, Pettitt’s test allowed us to classify the runners in two categories: the “fallers” who have a slightly statistically heterogeneous speed, and the “non-fallers” who have a homogeneous speed during the whole run.

We used the Pearson’s correlation coefficient to correlate the performance with the racing strategy (asymmetry of speed), the speed decrease (%), and the cardiac drift (cardiac cost increase). We then determined the significance level α = 0.05 for the interpretation of the statistical tests used. The entirety of the study has been conducted with the software XLSTAT^®^ version 2019, developed by the Adinsoft society. All the results are presented with mean ± standard deviation. At the end, we can also use the coefficient of variation, another speed race characteristic of speed time series (CV, i.e., standard deviation/mean%) that appears to be an easy and high marker of the difference of running strategy between the two groups.

## Results

We started with looking for a trend in the speed time series (by Kendall’s non-parametric rank correlation coefficient) and confirmed that almost 80% of the 280 marathoners (77%) had a decreasing trend in speed data. Then, we compared this group (*n* = 215) that we named the “fallers” with the group of the “non-faller” runners (*n* = 65) who did not have a significant speed decrease.

### The Difference of Performance Between the Fallers and Non-fallers’ Group

The ANOVA test showed that the fallers’ group had a significantly lower performance (3 h01:42 s ± 18:10 s min vs. 2 h54 min09 s ± 17 min 13 s, *F* = 15, *p* = 0.006) than the non-fallers.

### The Difference of Speed Strategy Between the Two Groups

All the marathoners ran in “positive split,” that is to say, with a speed decrease trend. However, the fallers’ group had a significantly higher speed decrease than those of the non-fallers’ group (-0.49 vs. -0.25 for the Kendall’s tau in the fallers’ vs. non-fallers’ group, *F* = 15, *p* = 0.0001).

**FIGURE 1:**
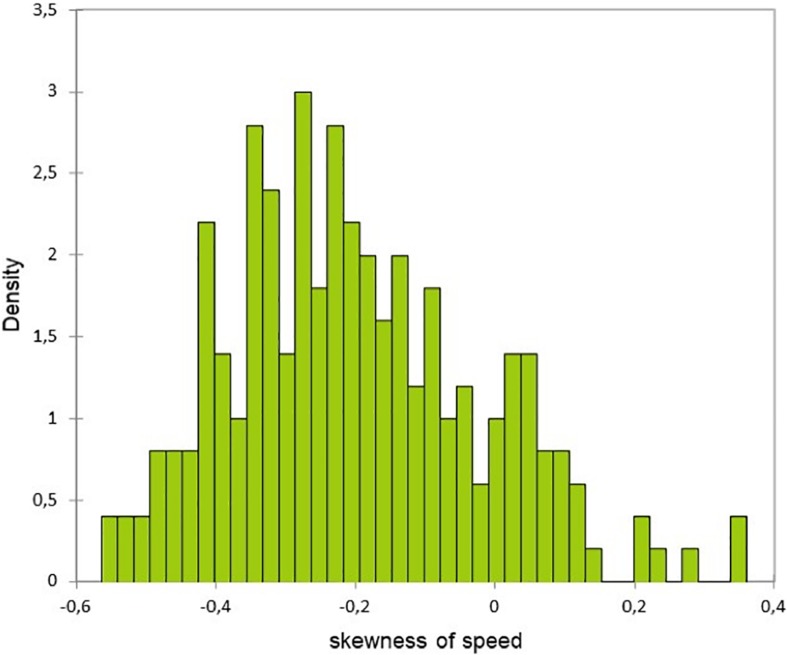
Distribution of Pearson’s asymmetry of speed for the faller group.

**FIGURE 2:**
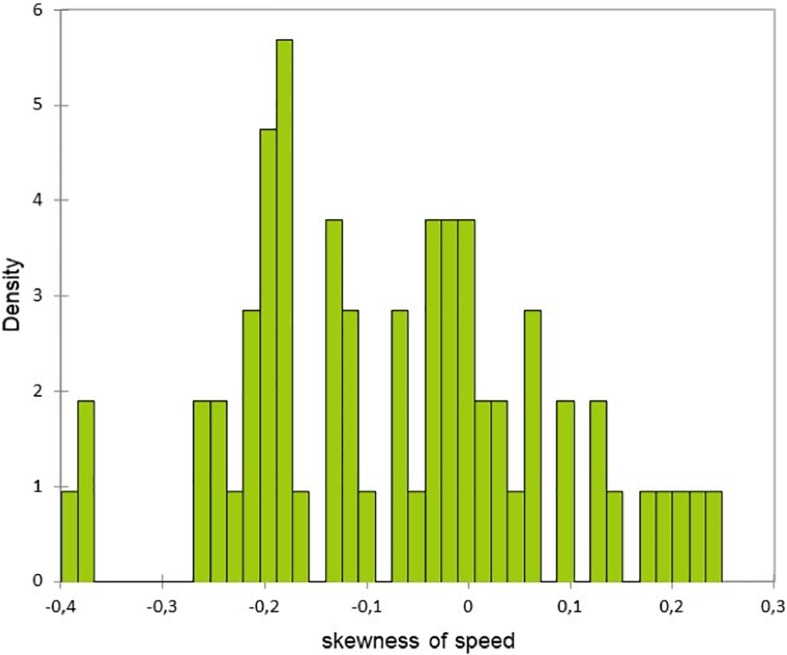
Distribution of Pearson’s asymmetry of speed for the non-faller group.

**FIGURE 3:**
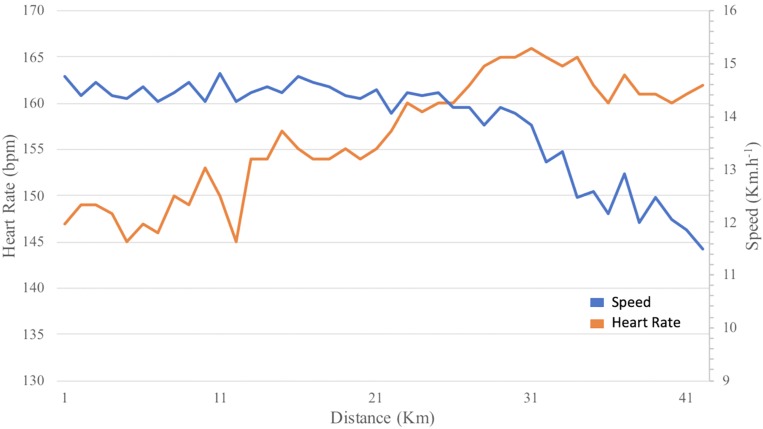
Plot of average change of state speed (blue) (km h^–1^) and heart rate (orange) (bpm) on the marathon.

In addition to this difference of the speed time course, the two groups had different speed race distribution. Indeed, the asymmetry indicator of the faller group runners’ speed was negative (Figure 1) in contrast with the non-faller’s group whose speed distribution was normal (Figure 2) (*F* = 28, *p* < 0.0001 and *F* = 11, *p* = 0.001, for speed and HR, respectively). Hence, the fallers’ group average speed was below their median speed, running 56% of the 42,195 km above their average speed. Then, they hit “the wall” as their speed falls sharply at the 26th km (Figure 3). However, interestingly, we can underline the normality of the cardiac cost distribution in both groups (*F* = 2.6, *p* = 0.1). It means that, whichever their speed profile, all the marathoners ran 50% of the time above and below their average cardiac cost.

### The Correlation Between Speed Strategy and Marathon Performance

**FIGURE 4:**
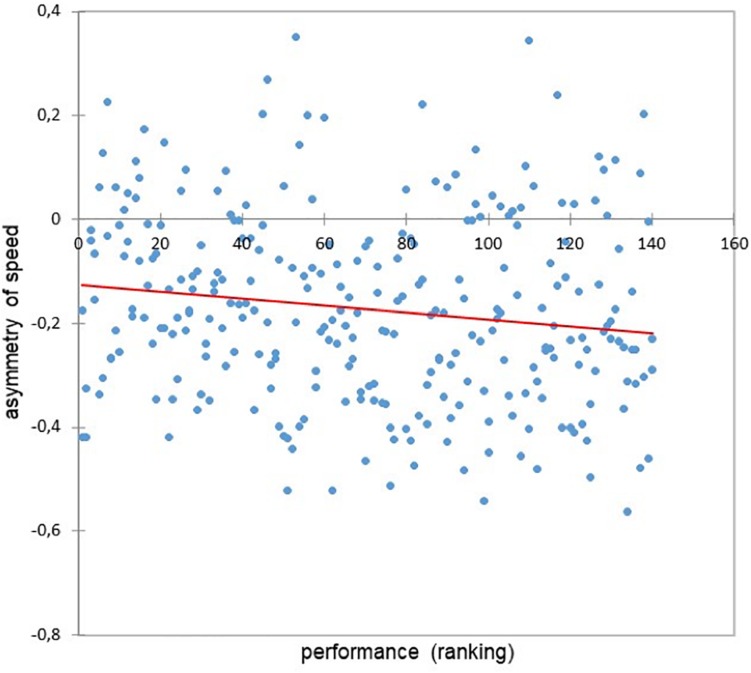
Relationship between performance and the asymmetry of speed (*r* = −0.15, *p* = 0.018).

The running asymmetry was significantly correlated with the performance (*r* = −0.15, *p* = 0.018) (Figure 4).

### The Difference of Heart Rate and Cardiac Cost Time Courses Between the Fallers’ and Non-fallers’ Group

The heart rate increase between the first and last 4 km of the marathon was not significantly different between the two groups (*F* = 2.0, *p* = 0.15) and was not correlated with the performance (*r* = 0.01, *p* = 0.80). Given that the non-fallers did not decrease their speed, their heart rate increased significantly more than in the fallers’ group (0.56 vs. 0.29, respectively, *F* = 14, *p* = 0.002). When we indexed the heart rate by the speed, we saw that in the fallers’ group, the cardiac cost increased significantly at the 26th km, as the speed did (Figure 3), and hence, the cardiac drift was then mainly associated with the speed decrease. Indeed, the cardiac cost increase between these first and last 4-km sections was highly correlated with marathon performance (*r* = 0.18, *p* = 0.002). Furthermore, we showed that marathon performance was correlated with the amplitude of the cardiac drift but not with that of the increase in the heart rate (*r* = 0.01, *p* = 0.80).

### The Relationship Between Heart Rate and Cardiac Cost Time Course and the Marathon Performance

**FIGURE 5:**
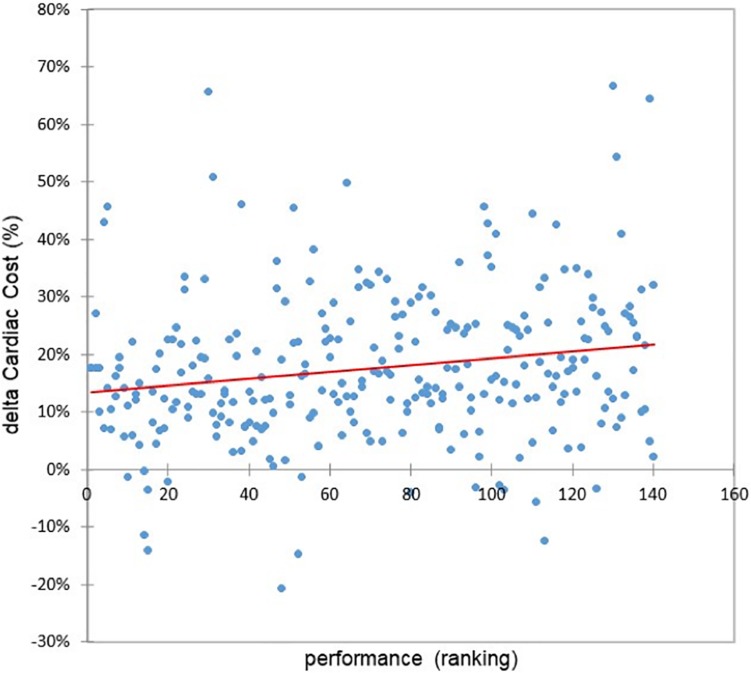
Relationship between delta cardiac cost (%) and the performance (*r* = 0.28, *p* = 0.0018).

**FIGURE 6:**
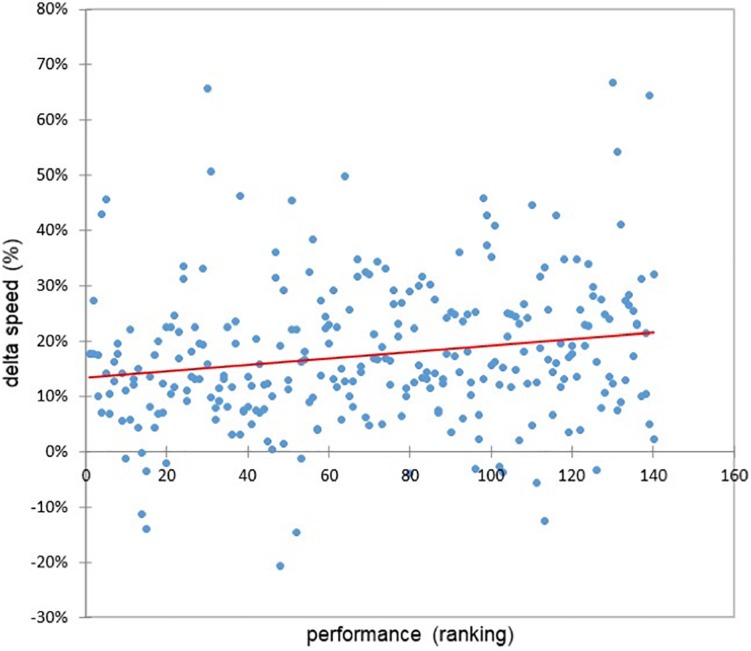
Relationship between delta speed (%) and the performance (*r* = −0.19, *p* = 0.001).

The increase in cardiac cost (cardiac drift) between the first and last 4-km parts of the marathon was highly correlated with performance (*r* = 0.28, *p* = 0.0018) (Figure 5). Indeed, this higher speed decrease (10% vs. 2%, *F* = 54, *p* < 0.0001) in the fallers’ group between the first and last 4-km parts of the marathon was highly inversely correlated with performance (*r* = −0.19, *p* = 0.001) (Figure 6).

### The Coefficients of Variation of Speed, Heart Rate, and Cardiac Cost Are High Markers of Difference of Running Strategy and Performance

**FIGURE 7:**
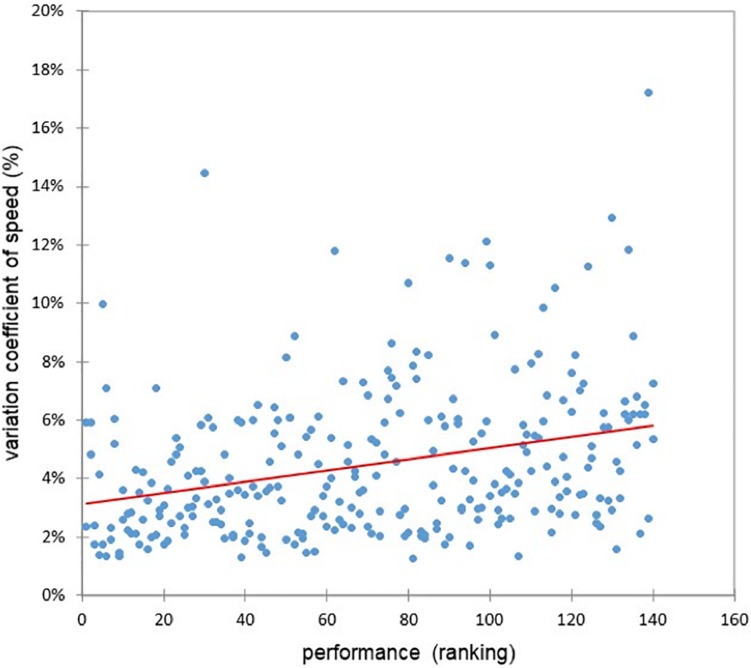
Relationship between the coefficient of variation of speed and the performance (*r* = 0.30, *p* < 0.0001).

**FIGURE 8:**
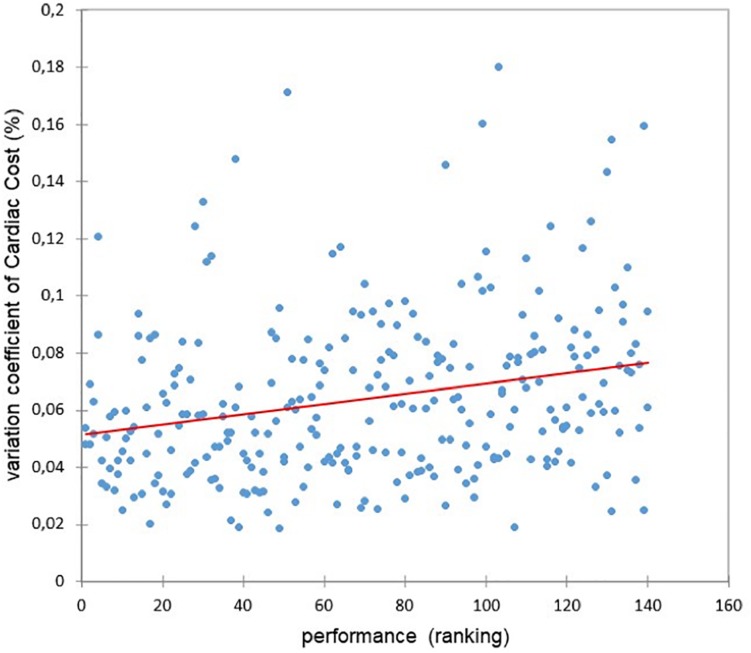
Relationship between the coefficient of variation of cardiac cost and the performance (*r* = 0.25, *p* < 0.0001).

The coefficient of speed variation (CV) was highly different between the two groups (5.0 vs. 2.9%, *F* = 36, *p* < 0.0001) and was highly correlated with performance (*r* = 0.30, *p* < 0.0001) (Figure 7). The coefficient of heart rate variation was also significantly higher in fallers (CV and *F* = *p*) than non-fallers but was not correlated with performance (*r* = 0.03, *p* = 0.63). However, the cardiac cost’s CV was significantly higher in the fallers’ than in the non-fallers’ group (*F* = 36, *p* < 0.0001) and correlated with performance (*r* = 0.25, *p* < 0.0001) (Figure 8).

## Discussion

We also remarked that, in the fallers’ group, the heart rate started to increase already at the half marathon, before the high-speed drop, which only appeared at the 26th km (Figure 3). This means that this significant sensitive HR drift was probably one cause of the exercise intensity speed downregulation. The heart rate started to increase already at the half marathon, but the cardiac drift, i.e., the cardiac cost increase, significantly appeared at the 26th km following the speed dynamics (Figure 3). This means that the heart rate increases before the speed decrease, which could be a signal of cardiac strain for pacing (Tyler, 2019, p. 20).

The main finding of the present study was that the racing strategy affects the sub-elite marathoner’s cardiac drift and performance.

Other important results have been achieved: (i) two groups of marathon runners can be distinguished according to their speed distribution and time series: the group of runners who have a drop in speed (77%) (called “speed fallers”) and those (group 2) who maintained their speed at the finish; (ii) speed fallers had a significantly higher cardiac drift and lower performance than non-fallers; (iii) cardiac drift was correlated with performance. Therefore, this study showed that the running strategy influences both performance and cardiac drift that covaries.

Most of the marathoners hit the marathon wall.

While the minority of these recreational (around 3 h) runners were able to sustain a constant speed with a low coefficient of variation until arrival, the great majority of them (3/4) “hit the marathon wall” at the 26th km. Indeed, in accordance with prior studies (Alvarez-Ramirez and Rodriguez, 2006; Billat V.L. et al., 2009; Wesfreid and Billat, 2009; Haney and Mercer, 2011; Renfree and St Clair Gibson, 2013), a change of the fractal scaling of the heart rate and speed in a marathon race has been detected, showing evidence of the significant effect of fatigue induced by such long and intensive exercise on the heart rate and speed variability (Wesfreid and Billat, 2009; Billat et al., 2012). Indeed, marathon elicits a high fraction of VO2max (70–90%), which has been estimated to be between 70 and 90% of VO2max according to the running economy at the marathon average speed (Costill, 1972; Sjödin and Svedenhag, 1985; Billat et al., 2001). This zone corresponds to subcritical speed, the one at which VO2 is not any more at a steady state and shows a drift until VO2max. Critical speed has been shown to be better correlated with marathon performance than VO2max or the ventilatory threshold (Billat et al., 1995; Florence and Weir, 1997). However, when real measures are performed during the real marathon race as did the excellent experiment of Maron et al. (1976), we can see that 100% of VO2max was elicited at the 26th km in one of the two good level marathoners (2 h30 min). Indeed, the speed is not constant and even more the oxygen cost of running per kilometer. This is due to the recruitment of supplementary fiber type II for compensating the muscular fatigue and strength responsible for losing elastic energy recoiling during the race (Gazeau et al., 1997; Slawinski and Billat, 2004; Rapoport, 2010). One has, indeed, considered that the ultimate limit to marathon performance might be dictated by the limits of running economy and a recruitment of the running musculature with a pattern that minimizes fatigue, possibly by spreading the work over many neurons (Coyle, 2007).

Running strategy must be integrated as a factor of marathon performance.

Indeed, marathon performance is explained not only by energetic factors but also by the running strategy that deserves more and more attention (Erdmann and Lipinska, 2013; Santos-Lozano et al., 2014; Renfree and Casado, 2018). The majority of marathoners, who were already experimented on, considered their performance given that our population average speed was around 3 h00 min, which was considered to be the Grail by marathon runners who wish to qualify for the historic Boston Marathon. We can also notice that this lack of stability of the running speed makes it more difficult to estimate the final time when speed beyond the half marathon is not available. However, the Massachusetts Institute of Technology mathematicians proposed a model to estimate the final time after the 2013 attack that prevented 6,000 marathon runners from reaching the finish line (Hammerling et al., 2014). A computational study has demonstrated that it was possible to predict the distance at which runners will exhaust their glycogen stores as a function of running intensity (Rapoport, 2010). They integrated several physiological variables including the muscle mass distribution, liver and muscle glycogen densities, and running speed as a fraction of aerobic capacity, i.e., the velocity at VO2max (Rapoport, 2010). They already have (2010) in mind shedding the physiological principle light on important standards in marathon that until now have remained empirically defined the qualifying times for Boston Marathon (Rapoport, 2010).

However, beyond performance prediction based on such anaerobic threshold (Rhodes and McKenzie, 1984; Loftin et al., 2007), critical speed (Florence and Weir, 1997), and VO2max (Billat et al., 2001) measurements, the fundamental question to solve now is how to give a better understanding of the rationale of speed control during the race (Coyle, 2007).

The necessity to have an interdisciplinary approach of the complexity of marathon pacing strategy.

This can be achieved thanks to an interdisciplinary approach crossing disciplining as psychology, neuroscience, physiology, physics, and mathematics allowing one to think the speed, heart rate, and other signals registered during the marathon in an entropy model control as a measure for non-stationary signals (Bollt et al., 2009).

Mathematics and statistics already allowed us to better describe the kinematics of running tactics (Erdmann and Lipinska, 2013). Erdmann and Lipińska (2006) investigated marathon running at the highest competitive level by examining the velocity distribution during marathon running. To illustrate the difficulty of targeting an appropriate speed on the first 5 km, they gave us an example: a female runner (in Berlin Marathon 2002) who attempted to break the world record running the first 5 km at a mean velocity higher than 5.00 m/s. This was too fast since it gradually decreased in the course of the race, resulting in a lower velocity during the second part of the run than during the first, as observed in 77% of our 280 recreational marathon runners.

This shows that the choice of the right speed on the first kilometer is not reserved to recreational runners, but is also a problematic and probably the major limiting factor now for sub-2 h marathon record. Even if, in terms of mechanical and steering approaches’ point of view, a long-distance run should be performed at a steady velocity (Foster et al., 1994; Maroński, 1996; Rapoport, 2010), stated that all deviations from the steady velocity should be within ± 2%, which was not the case even for the best performance. We can underline that it was the case for our 65 runners in the non-faller group who had a speed coefficient of variation of 2.9%, which was much lower than the coefficient of speed variation than the fallers (5%) and much lower than other less performer recreational runners (4 h).

It may also be possible to use the Talk Test (Foster et al., 2009) for calibrating the marathon speed at the condition to use the first stage before the one at which the runner cannot anymore declare to be able to “speak comfortably” during a Balke (incremental test). However, the Talk Test must be applied on real long event given that the heart rate response during long endurance event may be systematically higher (e.g., cardiovascular drift) than predicted from the Talk Test incremental exercise (Foster et al., 2008). That is why, by precaution, the speed at the last comfortable speaking one is recommended if the runner wants to use an incremental test such as the University Montréal Track Test (UMTT) currently used by marathoners for estimating their VO2max and then their marathon pace (Léger and Boucher, 1980).

We proposed, and another research group validated, a new field test based on a self-pace approach for estimating the ventilatory and VO2max speed. Indeed, if we want to reconnect the runner with himself, the goal of training would now be to self-pace-train and then run the race only with the rate of perception of exhaustion, calibrated prior by a self-pace test as the Rabit test recently validated, giving a biofeedback of the fractional utilization of VO2max for a given RPE value. This feeling of integrated approach of pacing allows avoiding the question of the cardiac drift for a given speed. Before reaching this faculty, we can suggest to use card cost, which has been reported to be maintained during the marathon in both groups. Indeed, such biofeedback could be recommended by mobile phone application and could be implemented in the already sophisticated cardio-frequency meter of GPS watches widely adopted by runners who use it only for controlling their schedule running times during the marathon.

Indeed, the increase in the heart rate, which appears in both groups at half marathon (5 km before the sharp speed drop in the fallers’ group), probably induces a higher cardiovascular strain resulting in a higher rating of perceived exertion. Therefore, downregulation in speed occurs as a result of increased perceived exertion, which, in turn, is a product of thermal comfort/discomfort and cardiovascular strain due to the link between ratings of perceived exertion and cardiovascular strain (Tyler, 2019) as in marathon (Cheuvront and Haymes, 2001; Billat et al., 2012; Faude and Donath, 2016). This downregulation allowing heart rate increase while the speed is maintained could be a factor of marathon performance, and the knowledge of its threshold increase tolerance for one runner could be a major indicator of the “sensory tolerance” limit proposed as a hypothetical construct determining exercise performance by Hureau et al. (2016) whichever the cause (Thompson, 2006).

Thus, to address the question of cardiac drift in marathon, which is very sensitive to running strategy, we recommend to utilize the cardiac cost, which takes into account the running speed. In conclusion, this study suggests that the amatory runners should not control their speed but rather choose a self-pace strategy keeping the GPS for biofeedback after the race. For instance, small speed variation in a range defined for each runner according to his energetics profile as the speed reserve between the maximal speed and the critical speed (Fukuba and Whipp, 1999; Renoux et al., 1999) could be used to allow a kind of intermittent exercise modality, which has been reported to prevent cardiovascular drift (Colakoglu et al., 2018). The cardiovascular drift has been reported to be highly correlated with the reduced maximal oxygen (Wingo et al., 2005). Therefore, and it may be then possible, that, as during the Mont-Blanc ascent where the alpinists decreased their accessional speed in such a way that they maintained their fractional use of VO2max equal to 75% (Billat et al., 2010), the marathoners adjust their pace for staying at the same fractional utilization of VO2max even after the increase in the cardiac drift. Indeed, the heart rate increase for a given speed represents an increase in the double product, which is an index of the myocardial VO2 (MVO2) increase (Mudge et al., 1979). This means that the respective skeletal and cardiac muscle VO2 could be changed and given that, in addition, the global VO2max during the race has been shown to be limited by the loss of power (Billat et al., 2013), this could force the runner to highly decrease his or her speed during the marathon (Billat et al., 2012).

### Limitation of This Study

However, a covariance between marathon performance and cardiac drift is not a causality since this was a cross-sectional and not a longitudinal study. Further analysis using data from the same runner to compare a successful marathon with an unsuccessful one will allow further knowledge of the prescription on the optimal running strategy based on the runner’s cardiac drift. The main limitations of this study concern the inability to control the physical conditions of the race (slope of the land, wind speed, etc.) and of the subjects (integration, supplementation, hydration, caffeine, cardiotonic substances, etc.).

## Conclusion

The increasing volume of data split available on running community websites allowed a recent study to investigate a marathon running pacing strategy for various levels of performance. It is our scientific responsibility to use this database in addition to continuing to apply experimental protocols to better understand personal runners’ optimal way, especially on such popular and intensive exercises such as marathon. Here we show that the use of cardiac cost as an objective tool for targeting marathon pace avoiding or at least minimizing hitting the wall could be the first step for learning how to self-pace long-distance run, which gathers almost all the metabolic and psychical limiting factors defining a sensory tolerance limit.

The perspective scope of future studies would be to include the cardiac cost in the analysis of the ability of runners to accurately maintain their pre-race target time and compare pacing and perceived exertion (RPE) of different groups of athletes according to how close they were to their predicted time as recently proposed by Piacentini et al. (2019).

## Data Availability Statement

The datasets generated for this study are available on request to the corresponding author.

## Ethics Statement

The study was performed in accordance with the Polish law and was evaluated by the Bioethical Committee at the Jerzy Kukuczka Academy of Physical Education in Katowice, which granted official approval for the research (KB/47/17). The study was conducted in conformity with the Declaration of Helsinki. As online surveys or questionnaires do not require the completion of a separate participant information sheet or consent form, completion of the survey was deemed to constitute informed consent.

## Author Contributions

VB and MC contributed and conceived the study. VB, MC, and J-RP designed the study and drafted the manuscript. MC collected, analyzed, and interpreted the data. All authors revised the manuscript and approved the final version.

## Conflict of Interest

The authors declare that the research was conducted in the absence of any commercial or financial relationships that could be construed as a potential conflict of interest.
